# Effect of once-a-day milk feeding on behavior and growth performance of pre-weaning calves

**DOI:** 10.5713/ab.23.0073

**Published:** 2023-08-23

**Authors:** Syed Husnain Mushtaq, Danish Hussain, Hifz-ul-Rahman, Muhammad Naveed-ul-Haque, Nisar Ahmad, Ahmad Azeem Sardar, Ghazanfar Ali Chishti

**Affiliations:** 1 Department of Livestock Production, University of Veterinary and Animal Sciences, Pattoki 55300, Pakistan; 2 Department of Animal Nutrition, University of Veterinary and Animal Sciences, Pattoki 55300, Pakistan

**Keywords:** Behavior, Calves, Growth, Milk Feeding Frequency

## Abstract

**Objective:**

The objectives of the present study were to evaluate the effects of once-a-day milk feeding on growth performance and routine behavior of preweaning dairy calves.

**Methods:**

At 22nd day of age, twenty-four Holstein calves were randomly assigned to one of two treatment groups (n = 12/treatment) based on milk feeding frequency (MF): i) 3 L of milk feeding two times a day; ii) 6 L of milk feeding once a day. The milk feeding amount was reduced to half for all calves between 56 and 60 days of age and weaning was done at 60 days of age. To determine the increase in weight and structural measurements, each calf was weighed and measured at 3 weeks of age and then at weaning. The daily behavioral activity of each calf was assessed from the 22nd day of age till weaning (60th day of age) through Nederlandsche Apparatenfabriek (NEDAP) software providing real-time data through a logger fitted on the calf’s foot.

**Results:**

There was no interaction (p≥0.17) between MF and sex of the calves for routine behavioral parameters, body weight and structural measurements. Similarly, there was no effect of MF on routine behavioral parameters, body weight and structural measurements. However, the sex of the calves affected body weight gain in calves. Male calves had 27% greater total body weight and average daily gain than female calves. There was no effect of the sex of the calves on behavioral measurements. Collectively, in the current study, no negative effects of a once-a-day milk feeding regimen were found on routine behavioral and growth parameters of preweaning calves in group housing.

**Conclusion:**

Once-a-day milk feeding can be safely adopted in preweaning calves from 22nd day of age.

## INTRODUCTION

Heifer rearing is critical for the future of a dairy operation. Among the different phases of heifer rearing, preweaning phase is the most expensive and critical [1]. In the preweaning phase, calves are at the highest risk of gastrointestinal and respiratory infections. Most of the calf mortality occurs during the preweaning period. Monitoring the routine behavior of calves can help dairy farmers decrease calf mortality and increase their growth performance through early diagnosis of pathological conditions [2,3]. Technology can be very helpful in monitoring calf behaviors in large dairy herds. It can reduce the cost involved in the treatment and management of calves. In preweaning calves once a day milk feeding can also decrease management costs.

In the preweaning phase, labor is the second-largest expense accounting for 13.8% of the total rearing cost [4]. Most of the labor cost (in terms of time) is spent on milk-feeding preweaning calves [5].

Different milk feeding frequencies have been used in preweaning calves [5,6]. Although currently, twice a day milk feeding is the most common practice [7]. Once day milk has the potential to reduce the labor cost by 40% without affecting calf health and growth [8]. Studies comparing once versus twice a day milk feeding regimens did not find any negative implications of once-a-day milk feeding on calf average daily gain (ADG), feed intake, health, and structural development [5,9,10].

Monitoring the routine behavior of calves can help in assessing the welfare status of calves. Routine behaviors of calves i.e lying time, lying bouts and step activity can be affected by various stressful conditions. Bacterial infections increase lying time, decrease step activity and lying bouts of calves [11]. Heat stress also decreases lying bouts while, pain due to castration decreases the number of steps and lying time in calves [12]. Changes in routine behavior parameters of calves can provide a good indication of the health and welfare of calves. Currently, there is little to no information about the influence of once-a-day milk feeding on the behavior of preweaning dairy calves. The objectives of the present study were to evaluate the effect of once-a-day milk feeding on weight gain, structural development (including heart girth and withers height) and behavior of preweaning calves. It was hypothesized that the growth performance, routine behavior, and structural development will not be different for calves fed milk once a day versus calves fed twice a day; thereby indicating once a day milk feeding can be a replacement for twice a day milk feeding in modern dairy farming.

## MATERIALS AND METHODS

### Experimental station

This study was conducted at Training and Research Demonstration Farm (T&RDF), University of Veterinary and Animal Sciences (UVAS), Ravi Campus, Pakistan. All the experimental procedures were performed in compliance with the institutional guidelines of the ethical review committee of UVAS (No. DR/287).

### Experimental design

Twenty-four new-born Holstein calves (males = 8, females = 16; birth weight = 35.10±4.41 kg) from T&RDF were enrolled in this study. All calves were fed 3 L of good quality colostrum within 3 hours after birth. To ensure the successful transfer of passive immunity, plasma total protein levels (as a measure of immunoglobulins) of calves were assessed using an optical refractometer (Fisherbrand Analog Refractometer). All calves had plasma total protein levels greater than 5.5 g/dL (6.0±0.29). After colostrum feeding, calves were kept in a single group, having both covered (6.1×9.8 m) and open (13.1 ×27.4 m) space, bedded with sand, and ventilated both naturally and mechanically with industrial fans (Model FS-75; Bilal Electronics, Lahore, Pakistan; blade length: 60 cm, with 15 cm). Each calf had a minimum of 2.5 m^2^ covered and a 15 m^2^ open area. Up to three weeks of age, all calves were fed 6 litres milk volumes in two equal feedings morning and evening. Buckets were used for feeding milk to all calves. Milk was heated up to 37°C for 15 minutes in a mobile calf feeder (GEA Dairy Feed F4650; GEA Group Aktiengesellschaft, Düsseldorf, Germany). Upon 3 weeks of age (at 22nd day of age), calves were randomly assigned to one of two treatment groups (n = 12/treatment) based on milk feeding frequency; i) 3 L of milk feeding two times a day (2×) ii) 6 L of milk feeding once a day (1×).

The milk feeding amount was reduced to half for all calves between 56 and 60 days of age. Weaning was done at 60 days of age. Calves on the 2× treatment were fed milk at 7:00 h and 18:00 h daily. Calves on the 1× treatment were fed milk at 07:00 h only. Freshwater and a commercial calf starter were available for calves all the time. Starter samples were analyzed for dry matter, crude protein [13], and neutral detergent fiber [14]. Calf starter composed of 55% corn, 30% soybean meal, 10% wheat bran, 4% molasses and 1% mineral premix on dry matter basis sample, contained 92% dry matter, 18% crude protein, and 18.2% neutral detergent fiber respectively.

To determine the ADG, each calf was weighed after 4 h of morning liquid feeding at 3 weeks of age and then at weaning (60 days of age) with an electronic scale (B-TEK Weight Technology, Karachi, Pakistan | Sang D60 Calibration & Interfacing). Similarly, for structural development heart girth and wither height were measured for all calves at 3 weeks of age and then at weaning (60 days of age). Heart girth was measured using a measuring tape (Fiber tape GWF-1506) while wither height was determined using a long steel ruler.

The daily activity of each calf was assessed from 3 weeks of age till weaning (60 days of age) through Nederlandsche Apparatenfabriek (NEDAP) CowControl software (Nedap Livestock Management, Groenlo, Netherland) which provided real-time data through a logger fitted on the calf’s foot. Twenty-one calves were used in the behavior study due to the lack of loggers. Eleven calves from group 1× and ten calves from group 2× were used in the behavioral study. Daily data for total lying time (hours/day), number of lying bouts, number of standups and number of steps for each calf was obtained from CowControl software. Further, daily standing time for each calf was obtained by subtracting total lying time from 24 hours of the day. Average lying time/bout was assessed by dividing total lying time by the number of lying bouts in the day. Lying and standing time percentage of the day were also calculated from 24 hours of the day.

### Statistical analysis

Data were analyzed using the MIXED procedure of SAS (version 9.4; SAS Institute Inc., Cary, NC, USA). For a change in body growth measurements, initial and final body weight (BW), ADG, feed to gain ratio and average of routine behavior parameters, milk frequency (MF), calf sex, and interaction of MF and calf sex were tested as fixed effects, while the week of the study was included as a random effect. The week of the study was included in the model to account for seasonal changes because calves were enrolled in the study on a rolling basis.

For routine behavior parameters of calves measured over age, the effect of calf sex was not significant and was removed from the model. Milk frequency was included as fixed effects for all behavior parameters of calves repeated over age. The goodness of fit criteria based on second-order Akaike information criterion values were used to select covariance structures for repeated measures. Autoregressive (1) was used as covariance structures. Week of the study and calf were used as random factors for all variables repeated over age. The least-squares means are presented in tables. Significant differences were declared at p≤0.05. For significant results, multiple comparisons were made using Tukey-adjusted p-values.

## RESULTS

### Behavior

There was no interaction (p≥0.17) between MF and sex of the calves for total lying time (hours/day), lying time percentage, number of lying bouts (number/day), average lying time/bout, standing time (minutes/day), standing time percentage/day, number of standing bouts, average standing time/bout, and number of steps (number/day). Similarly, there was no effect of either MF (p≥0.21) and sex (p≥0.10) of the calves for all behavior parameters (Table 1).

**Figure 1:**
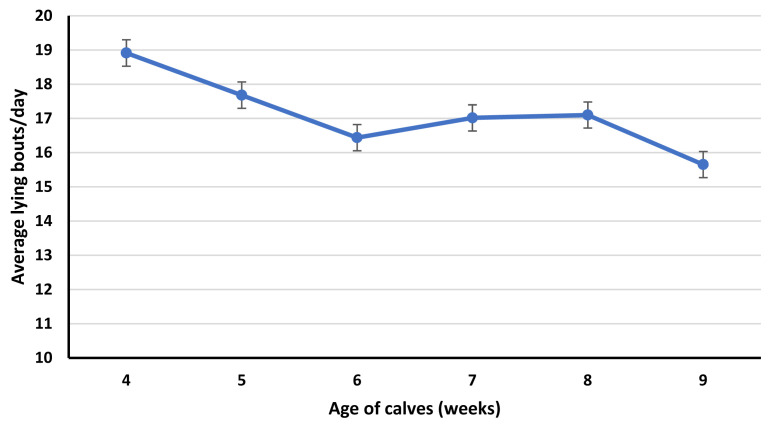
Effect of calf age (p≤0.01; from 4 to 9 weeks) on average lying bouts/d. The error bars reflect the standard error of the mean for average lying bouts/d for 4 to 9 weeks of age.

**Figure 2:**
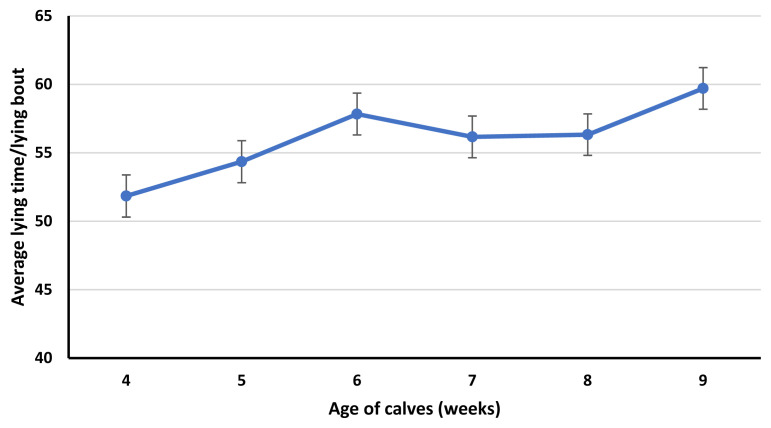
Effect of calf age (p≤0.01; from 4 to 9 weeks) on average daily lying time/lying bout. The error bars reflect the standard error of the mean for average daily lying time/lying bout for 4 to 9 weeks of age.

Lying bouts, average lying time/bout, standup counts and average standing time/standup were affected (p≤0.01) by the age of calves. Lying bouts were greater for calves in 4 weeks of age compared to calves in 6,7, 8 and 9 weeks of age. Similarly, calves in 5 and 8 weeks of age had greater lying bouts than the calves in 9 weeks of age (Figure 1). Average lying time per bout was less for calves in 4 weeks of age compared to calves in 6- and 9-weeks age (Figure 2).

**Figure 3:**
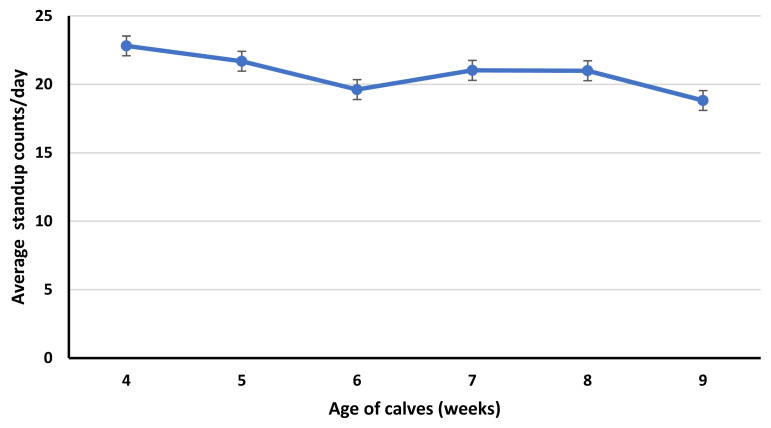
Effect of calf age (p≤0.01; from 4 to 9 weeks) on average daily standup counts. The error bars reflect the standard error of the mean for average daily standup counts.

**Figure 4:**
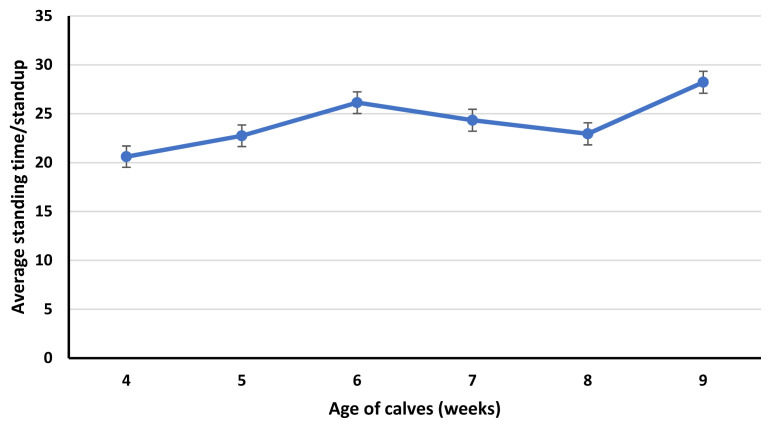
Effect of calf age (p≤0.01; from 4 to 9 weeks) on average daily standing time/standup. The error bars reflect the standard error of the mean for average daily standing time/standup.

Standup counts were greater for calves in 4 weeks of age compared to calves in 6 and 9 weeks of age. Similarly, calves in 5 weeks of age had greater standup counts than calves in 9 weeks of age (Figure 3). Average standing time per bout was less for calves in 4 weeks of age compared to calves in 6 and 9-weeks age. Moreover, the average standing time per bout was less for calves at 5 and 8 weeks of age compared to calves at 9 weeks of age (Figure 4). Total lying time (hours/day), lying time percentage/day, standing time percentage/day and number of steps (number/day) were not affected (p≥0.14) by the age of calves.

### Average daily gain and structural development

There was no interaction (p≥0.20) between MF and calf sex for all measurements of BW, wither height, and heart girth. Initial and final BW, wither height and heart girth were not affected by (p≥0.06) MF and sex of the calves. Similarly, total change and ADG in BW, wither height, and heart girth was not affected (p≥0.69) by MF of calves. However, total change and ADG of BW were affected by the calf sex. Male calves had 27% greater total BW and ADG than the female calves. There was no effect (p≥0.08) of sex on both total change and ADG of wither height and heart girth of calves (Table 2).

## DISCUSSION

Once-a-day milk feeding has been reported in preweaning calves since 1969 [9], yet its adaptation rate is less than 5% [7]. This may be due to the assumption that once-a-day milk feeding may negatively affect the behavior and welfare of calves. Rather, once-a-day milk feeding in calves tended to stimulate greater calf starter intake than the calves fed twice-a-day milk [6] which may help in greater rumen development and successful transition to a solid diet [15,16]. Once-a-day milk feeding presents an opportunity to ease calf management and reduce its rearing cost. This is the first study to evaluate the effect of once-a-day milk feeding on the routine behavior of Holstein calves.

Lying is important animal behavior, indicating its comfort and interaction with the environment [17]. In preweaning calves, group housing decreases lying time due to greater social interaction [18,19]. While bacterial infections can increase the lying time of preweaning calves to conserve their energy for the immune response against pathogens [20]. In the current study, there was no difference in lying time between once and twice a day milk-fed calves, indicating similar social interaction and exposure to pathogens for calves from both treatment groups. Lying time as a percentage of the day observed in our study is 6% and 7% less than reported by Chua et al [18] and Andrighetto et al [21], respectively in group-housed calves. Two and three calves were housed together by Chua et al [18] and Andrighetto et al [21], respectively, each calf having a space of 1.5 to 2 m^2^. In our study all calves were housed together, each calf having a minimum space of 17.5 m^2^, 8 to 10 times greater than by Chua et al [18] and Andrighetto et al [21]. Increased space may provide calves more opportunities to socially interact, thereby decreasing their lying time in the day. Therefore, the total lying time (951 min) observed in the current study, is slightly less than previously reported by Phillips [22] (972 min) and Swartz et al [23] (979 min) in group-housed calves. However, lying bouts per day in our study are greater (17 vs 11) than that reported by Phillips [22] in pair housed calves. This difference in lying bouts can also be attributed to the greater space available to the calves in our study. [24] has reported a 5.6% increase in lying bouts with each additional square meter of space allowance to adult bulls. Phillips [22] provided 10 times less space to each calf than that provided in our study. Swartz et al [23] reported similar lying bouts (19 vs 17) in preweaning calves to our study. However, they used only 5 calves and did not provide detail about the space available to each calf. Lying bouts decrease in calves due to respiratory diseases [25]. Similar lying bouts in once and twice a day milk-fed calves may suggest no difference in respiratory disease vulnerability of once-a-day milk-fed calves compared to twice-a-day milk-fed calves.

In our study, lying bouts and standup counts were affected by the age of calves. Both lying and standup counts of calves seem to decrease with the age of calves, probably because of the increased time spent in feeding calf starter [26]. In preweaning calves, calf starter intake increases with age. Lying bouts were greater for calves in 4 weeks of age compared to calves in 6, 7, 8, and 9 weeks of age. Similarly, calves in 5 and 8 weeks of age had greater lying bouts than calves at 9 weeks of age. While standup counts were greater for calves in 4 weeks of age compared to calves in 6 and 9 weeks of age. Similarly, calves in 5 weeks of age had greater standup counts than calves at 9 weeks of age. The decrease in lying bouts and standup counts in calves in 9 weeks of age is confounded by the age and reduced milk fed to calves. In week 9, milk volume for all calves was reduced to half to initiate weaning. Weaning affects the feeding and activity behavior of the calves, decreases lying bouts and increases feeding time [26]. As, lying bouts were less for calves in 9 weeks of age compared to calves in 4, 5 and 8 weeks of age. While standup counts were less for calves in 9 weeks of age compared to calves in 4 and 5 weeks of age. Similar to our study, Overvest et al [26] observed a decrease in lying bouts of weaning calves. This can be due to the greater time spent feeding calf starter compensating for the decrease in the liquid diet. Currently, there is little information available about the effect of weaning on the lying and standing behavior of calves.

Step activity can be an important behavioral marker to early diagnose pathological conditions in pre-weaning calves which are at high risk of several gastrointestinal and respiratory infections in the preweaning phase. There is very little information about the number of steps of preweaning calves. Our study is one of the few studies reporting step data for pre-weaned calves. The number of steps of preweaning calves reported by Swartz et al [23,25] were 2 times less than the steps of calves in our study. This difference in step activity can also be due to the greater availability of space for calves in the current study providing them the opportunity to have a greater walk. Step activity can decrease due to different stressors i.e. respiratory and gastrointestinal infections, pain due to castration and heat stress [27]. In this study, the step activity of once-day milk-fed calves was not different from the calves fed milk twice a day, indicating no extra stress for once-a-day milk-fed calves. Collectively, in the current study, no negative effects of once-a-day milk feeding regimen were found on the routine of behavior lying time, lying bouts, standing time, standing bouts and steps of preweaning calves in group housing, indicating this regimen may be successfully adopted to save labor cost especially in large dairy herds.

Similar to behavior parameters, no negative effect has been observed in preweaning ADG of calves fed milk once a day. Our results agree with [10,28,29], who also found no differences in preweaning ADG between calves fed milk once or twice a day. We found no published studies that reported differences between once and twice-daily feeding of milk to calves. Structural measurements which include withers height, and heart girth were the same in calves fed milk either once or twice a day. This result agrees with [28], who also did not report differences in withers height between calves fed milk once or twice a day. In our study, calf sex affected ADG. At 3 weeks of age, both males and females had no difference in their BW. However, at weaning male calves were 27% heavier than the female calves. This effect of sex on BW has also been reported previously [30–32]. Male calves grow more rapidly and reach a greater mature weight than the females because of the hormonal differences in their endocrinological and physiological functions and to selection pressure that was more intense on males than female calves [33]. This may also be because of the group housing with two sexes. Male calves may have dominated over female calves for calf starter intake, consuming greater starter and thereby resulting in greater weight than female calves. To the best of our knowledge, no study has compared the performances of male and female calves in group housing.

To conclude, in the current study, we found no negative effect of once-a-day milk feeding on behavior, growth, and structural development of Holstein calves. So once a day milk feeding can be safely adopted in group-housed dairy calves from 3 weeks of age.

## Figures and Tables

**Table 1:** Effect of frequency (1× and 2×) of milk feeding and gender on routine behavior of preweaning calves

Parameter	1×^1)^		2×^1)^		SEM	p-values^2)^		
		
	Male	Female	Male	Female		MF	S	MF×S
Lying time (min)	938.8	956.7	966.9	950.0	17.57	0.48	0.97	0.26
Lying time (%)	65.19	66.44	67.14	65.97	1.220	0.48	0.97	0.26
Lying bouts	17.67	17.09	16.89	17.34	0.572	0.59	0.90	0.30
Average lying time/bout (min)	54.00	56.51	57.46	55.35	1.873	0.48	0.90	0.17
Standing time (min)	501.2	483.2	473.1	490.0	17.57	0.48	0.97	0.26
Standing time (%)	34.81	33.56	32.86	34.03	1.220	0.48	0.97	0.26
Standup counts	21.22	21.00	19.72	21.41	1.186	0.59	0.47	0.36
Average standing time/standup (min)	24.40	23.74	24.43	23.48	1.731	0.94	0.59	0.92
Number of steps	3815	4307	4161	4768	378.8	0.21	0.10	0.85

SEM, standard error of the mean.

1) 1×, milk fed once a day; 2×, milk fed twice a day.

2) p-value represents the main effects of dietary treatments: MF, milk frequency (once vs twice a day); S, sex of calves (male vs female); MF×S, milk frequency by calf sex interaction.

**Table 2:** Effect of frequency (1× and 2×) of milk feeding and gender on body weight and growth measurements of preweaning calves

Parameter	1×^1)^		2×^1)^		SEM	p-values^2)^		
		
	Male	Female	Male	Female		MF	S	MF×S
Body weight (kg)								
Initial	45.9	49.4	47.7	46.7	2.15	0.79	0.46	0.20
Final	77.9	73.7	80.0	73.7	3.01	0.68	0.06	0.70
Change	32.1	23.6	30.7	25.9	2.91	0.87	0.02	0.47
ADG/d	0.82	0.61	0.79	0.66	0.074	0.87	0.02	0.47
Wither height (cm)								
Initial	83.0	84.6	82.8	82.1	1.32	0.25	0.67	0.34
Final	93.8	93.9	93.5	92.0	1.57	0.45	0.62	0.56
Change	11.1	8.60	10.2	9.69	1.01	0.89	0.08	0.23
ADG/d	0.28	0.22	0.26	0.25	0.026	0.89	0.08	0.23
Heart girth (cm)								
Initial	88.8	88.6	87.3	87.1	1.65	0.31	0.93	1.00
Final	100	99.6	99.5	97.9	1.40	0.37	0.42	0.61
Change	11.2	11.0	12.3	10.7	1.13	0.69	0.38	0.52
ADG/d	0.29	0.28	0.31	0.28	0.029	0.69	0.38	0.52

SEM, standard error of the mean; ADG, average daily gain.

1) 1×, milk fed once a day; 2×, milk fed twice a day.

2) p-value represents the main effects of dietary treatments: MF, milk frequency (once vs twice a day); S, sex of calves (male vs female); MF×S, milk frequency by calf sex interaction.
